# Difficult airway management and low Bispectral Index (BIS) in a patient with left Bochdalek congenital diaphragmatic hernia (CDH)

**DOI:** 10.1186/s12871-023-02140-x

**Published:** 2023-05-20

**Authors:** Ashraf A. Dahaba

**Affiliations:** Department of Anaesthesiology and Intensive Care Medicine, Suez Canal University, Ismailia, 41522 Egypt

**Keywords:** Bochdalek congenital diaphragmatic hernia, One lung anesthesia, Difficult airway management, Bioprosthetic Perceval aortic valve, Right lateral thoracotomy

## Abstract

**Background:**

Bochdalek congenital diaphragmatic hernia (CDH) is a developmental defect in the posterolateral diaphragm, allowing herniation of abdominal contents into the thorax causing mechanical compression of the developing lung parenchyma and lung hypoplasia. We describe a case of an adult patient with a Bochdalek hernia who underwent minimally invasive right thoracotomy Perceval bioprosthetic aortic valve replacement (AVR) requiring one-lung ventilation (OLV) on the side of the hernia. This is a complex and challenging case that brings up numerous thought-provoking anesthetic implications. To the best of our knowledge, a Pubmed search did not reveal any publication to date of difficult airway management in an adult patient with CDH.

**Case presentation:**

The first major problem encountered was patient’s crus habitus anatomical condition (exceedingly ventrally displaced trachea) Mallampati Class IV and Cormack-Lehane grade IV extremely difficult endotracheal intubation. Neither glottis nor epiglottis was visible on laryngoscopy; resulting in failed placement of the double-lumen endobronchial tube (DLT) following numerous attempts. The DLT was eventually placed via GlideScope videolaryngoscopy. Whereas the endobroncheal right lung block for left OLV was successfully placed using fiberopticscopy. The crus habitus encroached on OLV tidal volume by the cranially displaced ascending colon and left kidney. Anesthesia was maintained with remifentanil /sevoflurane; adjusted to maintain bispectral index (BIS) at 40–60. Digitally recorded BIS was 38–62 except when BIS precipitously declined to 14–38 (SR, suppression ratio < 10) for 25 min after termination of the cardiopulmonary bypass.

**Conclusions:**

We report a case essentially dealing with an anatomically distorted difficult airway in a patient with left Bochdalek CDH undergoing a complex AVR. We describe anesthetic difficulties and unforeseen issues encountered; such as an extremely difficult DLT placement.

**Clinical Implications**.

1. Bochdalek congenital diaphragmatic hernia (CDH) is a developmental defect in the posterolateral diaphragm; allowing herniation of abdominal contents into the thorax; causing mechanical compression of the developing lung parenchyma and lung hypoplasia.
2. This is a case essentially dealing with a difficult airway management in a patient with a left Bochdalek CDH, an anatomically distorted condition, undergoing one-lung anesthesia for a Perceval bioprosthetic aortic valve replacement (AVR).
3. We describe the anesthetic difficulties of this challenging case and the unforeseen issues encountered such as extremely difficult endotracheal intubation.

## Background

Bochdalek hernia, first described in 1754 by McCauley, was subsequently studied and named after the Czech pathologist Vincenz Alexander Bochdalek (1801–1883) [1]. Bochdalek hernias, a developmental defect in the posterolateral diaphragm, allowing herniation of abdominal contents into the thorax causing mechanical compression of the parenchyma of the developing lung and lung hypoplasia. Bochdalek hernias are a very rare form of diaphragmatic hernias. Manifesting symptoms typically tend to be respiratory symptoms. Symptomatic adults are rarely diagnosed, with the majority of cases incidentally discovered. The exact etiology is unknown, though both genetic and environmental components are plausible. In patients with Bochdalek hernia, the deformed lung will result in life-threatening lung compression. Bochdalek hernias are 85% more commonly occurring on the posterior left side (versus 15% occurring on the right side) [1].

We present a case report describing the care of an adult patient with a Bochdalek congenital diaphragmatic hernia [1] (CDH, left colon flexure and left kidney large retro-cardiac mass in the intrathoracic region) who underwent minimally invasive right thoracotomy bioprosthetic Perceval aortic valve replacement (AVR, CORCYM S.r.l., Saluggia, Italy) [2] for a massively calcified congenital bicuspid aortic valve that required one-lung ventilation (OLV) on the side of the hernia. Because patient was relatively young (late 50s); he decided against having the transaortic valve insertion long term anti-coagulants therapy. Here we describe the anesthetic difficulties encountered with this distorted anatomical condition, as all surgical challenges are already-published in another report fully describing the same case [2]. Perhaps anesthesiologists could gain some insightful aspects from this report.

## Case presentation

**Fig. 1:**
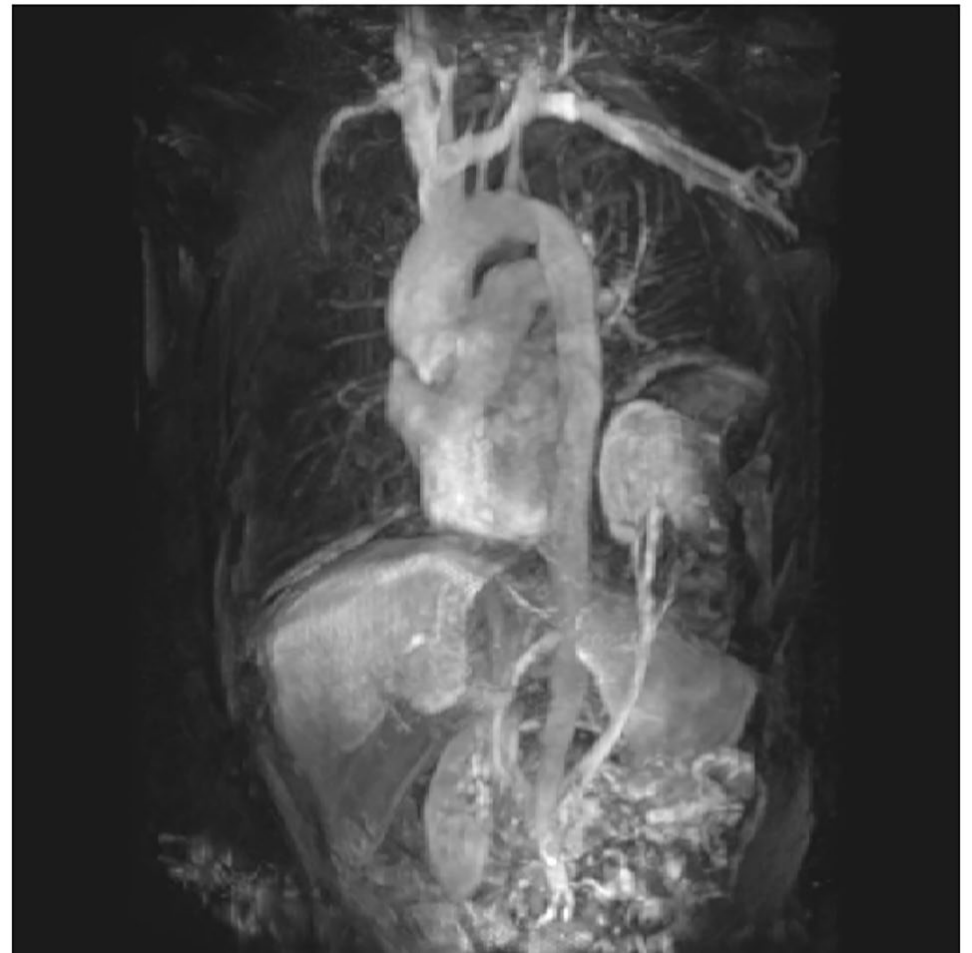
Magnetic resonance angiography. Left diaphragmatic crus habitus (comprising left kidney and left colon flexure Bochdalek congenital-diaphragmatic-hernia) is 67 mm displaced higher than the right diaphragmatic crus. The left ureter is 9 cm elongated and the left renal artery is also 9 cm retro-aortic elongated with two renal veins. Ascending aortic aneurism 47 mm

Patient gave a written informed consent that we can use all his real-time digitally recorded data. Dotarem 3D-contrast medium enhanced magnetic resonance angiography revealed a 47 mm aortic aneurism 5 cm cranial to aortic valve and a 16 mm coarctation of descending aorta at left subclavian artery outlet. Left diaphragmatic crus habitus (comprising left kidney and left colon flexure, Fig. 1) was 67 mm higher than the right diaphragmatic crus [2]. Left ureter was 9 cm elongated. Left renal artery was also 9 cm retro-aortic elongated with a double renal vein. Echocardiography showed 17 mm hypertrophy of the left ventricle.

Because our patient’s aortic aneurism was 4.7 cm, and the American College of Cardiology/American Heart Association (ACC/AHA) valvular heart disease guidelines recommend replacement of the ascending aorta when it reaches > 4.5 cm in a patient undergoing AVR; [3] different strategies for AVR, ascending aorta repair and contingency plans for failed OLV or oxygenation were all presented, planned and discussed.

Preoperative laryngeal examination revealed Mallampati Class IV anatomical condition of exceedingly ventrally displaced trachea, a condition that was not clearly visible in preoperative magnetic resonance imaging. The first major problem encountered was Cormack-Lehane grade IV extremely difficult endotracheal intubation. Following fentanyl 1–2 µg kg^− 1^, propofol 2–3 mg kg^− 1^ and rocuronium 100 mg for tracheal intubation, anesthesiologists documented in patient’s chart “limited laryngeal space because the epiglottis was extremely ventrally displaced”. Neither glottis nor epiglottis was visible on laryngoscopy resulting in failed placement of the double-lumen endotracheal tube (DLT) following numerous attempts. The DLT was eventually inserted via a GlideScope videolaryngoscopy. The endobroncheal right lung block for left OLV was successfully placed in the “extremely ventrally displaced trachea” using a fiberopticscope. The numerous intubation attempts resulted in laryngeal edema that required antihistaminic administration, but no apparent laryngeal injury or trauma.

The crus habitus encroached on OLV tidal volume by the cranially displaced ascending colon and left kidney. Skilled anesthesiologists successfully maintained acceptable arterial blood gases levels, adequate tidal volume ventilation and oxygenation (pressure control with volume guarantee 6–7 cmH_2_O PEEP, 50–100% FiO_2_, 140–279 mmHg PaO_2_, 98.9–99.9% SpO_2_ lung protection strategy).

A clear surgical strategy was devised based upon right anterior minimal invasive mini-thoracotomy approach [4] and a Perceval self-anchoring, self-expanding valve, designed for quick sutureless deployment allowing quick valve placement under direct vision [2]. The surgical strategy aim was maximal reduction of blood loss, surgical trauma, operative procedure time and intensive care unit (ICU) stay [4]. Multiple studies have demonstrated adverse effects of prolonged operative time; as *Ranucci et al.* [5] demonstrated 1.4% increase in severe cardiovascular morbidity for each additional minute of aortic cross clamping (ACC) time [5].

**Fig. 2:**
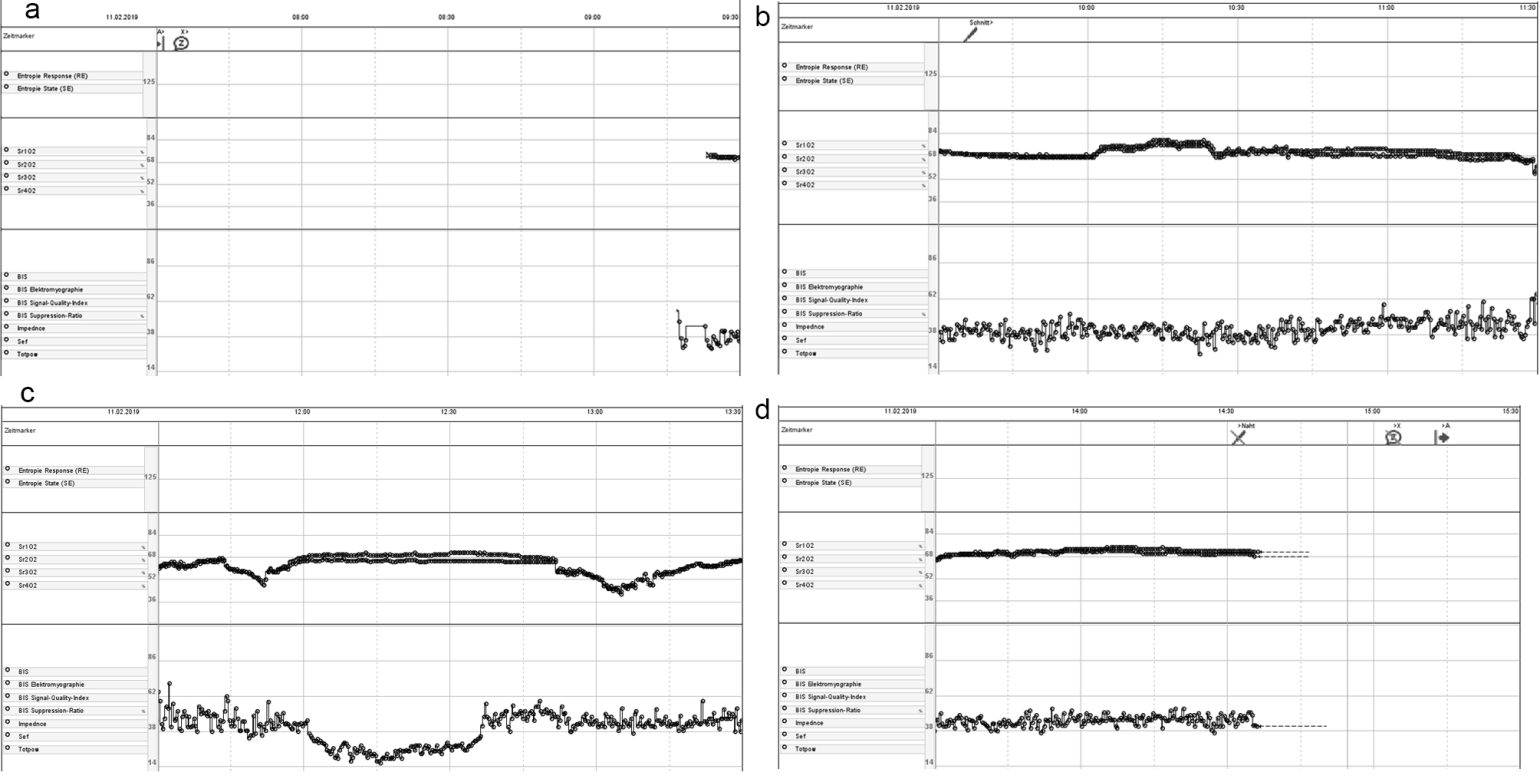
**a, b, c** and **d** Digitally recorded Bispectral index (BIS) during the entire anesthesia was between 38 and 62 except when BIS precipitously sustained a 25 min decline between 14 and 38 after the termination of cardiopulmonary bypass

Remifentanil /sevoflurane anesthesia was adjusted throughout the whole procedure to maintain bispectral index (BIS) at 60 − 40. Placement of vascular cannulae for transfemoral femoro-femoral cardiopulmonary bypass (CPB) was uneventful and a lateral right mini-thoracotomy incision successfully gained access to the desired surgical field. The annulus of the massively calcified 0.5 cm² aortic valve area (AVA) congenital bicuspid aortic valve was decalcification, and a sutureless self-expandable Perceval valve was placed. During the entire anesthesia, the digitally recorded BIS was 38–62 except when BIS precipitously sustained a decline to 38 − 14 (SR, suppression ratio < 10) for 25 min after the termination of the CPB when mean arterial pressure (MAP) dropped to 39 − 35 mm Hg under noradrenaline (0.06 mg ml^− 1^) 8–13 ml h^− 1^ continuous infusion (Fig. 2a, b, c and d).

Despite the laryngeal edema resulting from numerous intubation attempts; extubation was uneventful. Piritramide 30 mg was administered for postoperative analgesia. Patient spent one day in the ICU, one day in the Intermediate Care, was then transferred to the ward and had an uneventful post-operative course with a well-positioned aortic valve prosthesis.

## Discussion

This is a complex and challenging case that brings up numerous thought-provoking anesthetic implications. We present the anesthetic care of an adult patient with a Bochdalek hernia who underwent minimally invasive surgical AVR requiring OLV on the side of the hernia. To the best of our knowledge, a Pubmed search did not reveal any publication to date of difficult airway management in an adult patient with CDH. The skilled anesthesiologists successfully overcame a “severely ventrally displaced” tracheal intubation using GlideScope and fibro-optic techniques for the OLV. The GlideScope technique offers several advantages for difficult airway management of improved glottic view and intubation conditions in patients with a potentially difficult airway compared with a conventional direct laryngoscope. In simulated difficult airways, the use of GlideScope® and Airtraq® resulted in a lower score of difficult intubation than the King Vision™ [6]. Moreover, a randomized controlled study reported 100% success with GlideScope for DLT intubation better than other videolaryngoscopes [7]. Whereas using the GlideScope DLT intubation videolaryngoscopy results in milder cardiovascular response compared to classic direct laryngoscopy [8].

The CDH potentially landed the endoscopic surgical instrumentation in a distorted surgical field leaving little room for surgical adjustments to the originally planned anatomical position [2]. Experienced surgeons successfully navigated their approach and avoided potential injuries to the upward displaced colon flexure and left kidney [2]. Reduction of the length of procedure and surgical trauma depended on the use of Perceval sutureless bioprosthetic heart valve made of bovine pericardium leaflets designed for quick deployment, that does not entail a sewing ring hence resulting in vastly improved hemodynamics [2]. Although our patient had indeed a short ICU stay; our patient’s restricted surgical field resulted in a prolonged CPB 3 h duration re-cross clamp for AVR revision [2]. Thus regarding our initial aim of reducing the operative time, ACC duration, operative trauma and blood loss all proved to be not very beneficial and advantageous as this was not achieved.

After the termination of the CPB while MAP dropped to 39 − 35 mm Hg, the BIS indices declined to 38 − 14 that persisted for 25 min under noradrenaline continuous infusion. At anesthesia recovery no medications that were shown to alter the BIS monitoring such as flumazenil were administered [9]. BIS monitoring was shown to be a highly sensitive useful specific indicator for detecting cross-clamping decline in cerebral blood flow due to hypotension [10]. In 2 previous reports after the restoration of cardiac rhythm, the precipitous sustained BIS decline has been suggested as a clear indicator of cerebral ischemia during off-pump coronary artery bypass grafting when the heart was twisted for circumflex artery grafting, [11, 12] as our patient could have suffered from a twist of coronary artery during surgery.

We present a challenging case essentially dealing with the care of an adult patient with a left Bochdalek hernia retro-cardiac mass, comprising left colon flexure and left kidney who underwent minimally invasive AVR requiring DLT for OLV. This is a complex case that brings up numerous thought-provoking anesthetic implications as difficult airway management with GlideScope or hypotension after CPB to prevent cerebral ischemia low BIS values. It is certainly important for anesthesiologists to appreciate the unique anesthetic considerations presented in this case.

## Data Availability

Not applicable, as our manuscript does not contain any additional data other than reported data. Author declares that all relevant material are readily available.

## Abbreviations

BIS: Bispectral Index
CDH: congenital diaphragmatic hernia
AVR: aortic valve replacement
DLT: double-lumen endotracheal tube
OLV: one lung ventilation
ACC: American College of Cardiology
AHA: American Heart Association
ICU: intensive care unit
ACC: aortic cross clamping
CPB: cardiopulmonary bypass
AVA: aortic valve area
MAP: mean arterial pressure

## References

[1] Brown SR, Horton JD, Trivette E, Hofmann LJ, Johnson JM (2011). Bochdalek hernia in the adult: demographics, presentation, and surgical management. Hernia.

[2] Dahaba AA (2022). First case of a patient with left Bochdalek congenital diaphragmatic hernia and congenital bicuspid aortic valve stenosis undergoing Perceval bioprosthetic aortic valve replacement via mini-thoracotomy one-lung anesthesia. Asian J Surg.

[3] Otto CM, Nishimura RA, Bonow RO, Carabello BA, Erwin JP, Gentile F, Jneid H, Krieger EV, Mack M, McLeod C, O’Gara PT, Rigolin VH, Sundt TM, Thompson A, Toly C (2021). 2020 ACC/AHA Guideline for the management of patients with valvular heart disease: a report of the American College of Cardiology/American Heart Association Joint Committee on Clinical Practice Guidelines. Circulation.

[4] Murtuza B, Pepper JR, Stanbridge DR, Jones C, Rao C, Darzi A, Athanasiou T (2008). Minimal Access aortic valve replacement: is it worth it?. Ann Thorac Surg.

[5] Ranucci M, Frigiola A, Menicanti L, Castelvecchio S, de Vincentiis C, Pistuddi V (2012). Aortic crossclamp time, new prostheses, and outcome in aortic valve replacement. J Heart Valve Dis.

[6] El-Tahan MR, Al’ghamdi AA, Khidr AM, Gaarour IS (2016). Comparison of three videolaryngoscopes for double-lumen tubes intubation in simulated easy and difficult airways: a randomized trial. Minerva Anestesiol.

[7] El-Tahan MR, Khidr AM, Gaarour IS, Alshadwi SA, Alghamdi TM, Al’ghamdi A (2018). A comparison of 3 videolaryngoscopes for double-lumen tube intubation in humans by users with mixed experience: a randomized controlled study. J Cardiothorac Vasc Anesth.

[8] Wei W, Tian M (2016). Double-lumen tube intubation using video laryngoscopy causes a milder cardiovascular response compared to classic direct laryngoscopy. Pak J Med Sci.

[9] Dahaba AA, Bornemann H, Rehak PH, Wang G, Wu XM, Metzler H (2009). Effect of Flumazenil on Bispectral Index monitoring in unpremedicated patients. Anesthesiology.

[10] Dahaba AA, Xue JX, Hua Y, Liu QH, Xu GX, Liu YM, Meng XF, Zhao GG, Rehak PH, Metzler H (2010). The utility of using the Bispectral Index-Vista for detecting cross-clamping decline in cerebral blood flow velocity. Neurosurgery.

[11] Mourisse J, Booij L (2003). Bispectral index detects period of cerebral hypoperfusion during cardiopulmonary bypass. J Cardiothorac Vasc Anesth.

[12] Hemmerling TM, Olivier JF, Basile F, Le N, Prieto I (2005). Bispectral index as an indicator of cerebral hypoperfusion during off-pump coronary artery bypass grafting. Anesth Analg.

