# Correction: Difficult airway management and low bispectral index (BIS) in a patient with left Bochdalek congenital diaphragmatic hernia (CDH)

**DOI:** 10.1186/s12871-023-02247-1

**Published:** 2023-08-19

**Authors:** Ashraf A. Dahaba

**Affiliations:** Department of Anaesthesiology and Intensive Care Medicine, Suez Canal University, Ismailia, 41522 Egypt

**Correction: BMC Anesthesiol 23, 172 (2023)**

10.1186/s12871-023-02140-x

Following publication of the original article [1], the email address of the corresponding author has been updated from ashraf.dahaba@medunigraz.at to xiaozhaoy2012@163.com.
